# Joint predictability of health related quality of life and leisure time physical activity on mortality risk in people with diabetes

**DOI:** 10.1186/1471-2458-13-67

**Published:** 2013-01-24

**Authors:** Chia-Lin Li, Hsing-Yi Chang, Chih-Cheng Hsu, Jui-fen Rachel Lu, Hsin-Ling Fang

**Affiliations:** 1 Department of Health Care Management, College of Management, Chang Gung University, 259 Wen-Hwa 1st Road, Kwei-Shan, Tao-Yuan 333, Taiwan; 2 Healthy Aging Research Center, Chang Gung University, 259 Wen-Hwa 1st Road, Kwei-Shan, Tao-Yuan 333, Taiwan; 3 Division of Preventive Medicine and Health Service Research, Institute of Population Health Sciences, National Health Research Institutes, #35, Keyan Road, A3223, Zhunan town, Maoli 350, Taiwan

**Keywords:** Diabetes, Health related quality of life, Leisure time physical activity, Mortality, Taiwan

## Abstract

**Background:**

Reduced health related quality of life (HRQOL) has been associated with increased mortality in individuals with diabetes. In contrast, increased leisure time physical activity (LTPA) has been associated with reduced mortality. The aim of this study was to investigate the combined relationship of HRQOL and LTPA on mortality and whether high levels of LTPA are associated with reduced risk of mortality in adults with diabetes and inferior HRQOL.

**Methods:**

We analyzed data from a national sample of adults (18 years or older) with self-reported physician-diagnosed diabetes, who participated in the 2001 National Health Interview Survey in Taiwan (N = 797). A total of 701 participants had complete Short Form 36 (SF-36) and LTPA data and were followed from 2002 to 2008. Participants were divided into 3 groups based on their LTPA: (1) a regularly active group who reported 150 or more min/week of moderate-intensity activity; (2) an intermediately active group who reported engaging in LTPA but did not meet the criterion for the “regular” category; and (3) an inactive group who reported no LTPA. The physical component summary (PCS) and mental component summary (MCS) scores were dichotomised at the median (high vs. low) (PCS = 45.11; MCS = 47.91). Cox proportional-hazards models were used to investigate associations between baseline characteristics and mortality.

**Results:**

After 4,570 person-years of follow-up, 121 deaths were recorded and the crude mortality rate was 26.5 per 1,000 person-years. Both PCS scores and LTPA were significant predictors of mortality, whereas no significant relationship was observed between MCS and mortality. After adjustment for other factors, participants with low PCS who reported no LTPA had a hazard ratio (HR) for mortality of 4.49 (95% CI = [2.15-9.36]). However, participants with low PCS who were active (including intermediate and regular LTPA) had a HR for mortality of 1.36 (95% CI = [0.64-2.92]).

**Conclusions:**

Our results show a significantly increased mortality risk of diabetes associated with reduced HRQOL in individuals who report no LTPA. Engaging in LTPA may be associated with improved survival in participants with diabetes with poor self-rated physical health status.

## Background

Subjective assessment of health related quality of life (HRQOL) is an important health outcome not only because it captures a person’s perception of their overall health status, but also because it can inform resource allocation decisions. Recent research demonstrates that HRQOL is an independent predictor of mortality in people with diabetes [1-4]. Our previous research has also found that a self-perceived negative profile on the physical health domains of HRQOL in people with diabetes can predict those who are at higher risk for hospital admission even after taking into account known risk factors [5]. These findings highlight that the association between reduced HRQOL and further adverse outcomes has been understudied and that HRQOL has great potential value as a clinical marker that could be used to identify individuals with diabetes who are at higher risk of adverse outcomes and who therefore require more care.

HRQOL in people living with diabetes has been shown to be negatively influenced by diabetes-specific attributes, including higher glycosylated haemoglobin level, the presence of diabetes complications, and the severity of diabetes symptoms [6-10]. These diabetes-specific attributes are also known to be associated with an increased risk of mortality in people with diabetes [11-14]. Adequate physical activity is considered to be one of the most important self-care behaviours for glycemic control and the prevention of vascular events in people with diabetes [15]. Prior cross-sectional studies of adults with diabetes have demonstrated that increased leisure time physical activity (LTPA) is significantly associated with better HRQOL [16] and increased LTPA has been linked to a lower risk of mortality in individuals with diabetes [15,17]. However, prior studies exploring the relationship between HRQOL and mortality in individuals with diabetes have not controlled for LTPA. It is still not clear whether the relationship between reduced HRQOL and increased mortality remains among those individuals with diabetes who engage in higher levels of LTPA. Clarifying the association between LTPA and mortality in people with diabetes who have reduced HRQOL is particularly important because it may help in the design of intervention strategies.

In view of these considerations, we have analyzed data from a 7-year prospective study of a national sample of adults with diabetes in Taiwan. The aims of the present study were two-fold. First, we investigated whether HRQOL remained an independent predictor of mortality in adults with diabetes after controlling for LTPA. Second, we explored the combined associations of HRQOL and LTPA on mortality in order to examine whether higher levels of LTPA were associated with reduced risk of mortality in adults with diabetes with inferior HRQOL.

## Methods

### Study population

This was a prospective study involving participants in the 2001 National Health Interview Survey (NHIS) in Taiwan. The sample design of the NHIS has been described in detail previously [18,19]. In brief, a representative sample was drawn from the National Registry Database via multistage stratified sampling. The survey obtained ethical approval from the Institutional Review Board of the National Health Research Institutes. All study participants provided informed consent. The original sample comprising 22,121 participants (response rate; 94.2%), can be treated as a simple random sample (SRS). Out of 16,137 participants aged 18 years or older, 797 reported physician-diagnosed diabetes. The crude prevalence of diabetes of adults aged 18 years or older was therefore 4.94% (797/16137), which does not include individuals with undiagnosed diabetes. Thus, the prevalence of diabetes (4.94%) is likely to be underestimated and we may not be able to generalize our findings to individuals with undiagnosed diabetes. The agreement between self-reported physician-diagnosed diabetes and confirmed diabetes was very good [18]. Of these potential participants, we excluded 93 individuals with incomplete data regarding their health-related quality of life or leisure-time physical activity and 3 individuals who had died by the end of 2001, leaving 701 eligible participants for the analysis. The study cohort was followed until December 31, 2008, via personal identification numbers in the computerized data files of the National Register of Deaths. We compared the characteristics of study participants who were included (N = 701) versus those who were excluded due to incomplete data (N = 93) in order to assess the degree of respondent bias.

### Measures

This study used the Taiwanese version of the SF-36, which was validated and has been shown to have good psychometric properties in a representative nationwide sample [20]. The SF-36 questionnaire has 36 items divided into eight dimensions (i.e., physical function, role limitations due to physical health problems, bodily pain, general health perceptions, vitality, social function, role limitations due to emotional problems, and mental health). Lower scores indicate poorer HRQOLs. The physical component summary (PCS) and the mental component summary (MCS) are aggregated from z-score transformations of the eight dimensions and then standardized to a mean of 50 and a standard deviation of 10 [21].

In this study, participation in LTPA was assessed first by asking respondents to reply “yes” or “no” to the question: “Have you engaged in any kind of leisure activity in the previous two weeks?” Those individuals replying “yes” to the question were asked to categorize these activities (up to three) according to a list of predefined typical leisure activities in this population. These activities included walking leisurely, jogging or race walking, swimming, traditional Chinese exercise, sports, aerobics, folk dancing, bicycling, mountain climbing, weightlifting and walking up stairs. There was also an “other” option where participants could report any specific activities which were not in the list. For each activity, the respondents also indicated the frequency and duration (hours and minutes) of the activity in the 2 weeks prior to the interview. Measures of physical activity were modified from the Nutrition and Health Survey in Taiwan [22,23]. The internal consistency of the questions for major types of exercise was 0.88. The Kappa for the reported frequency and duration of activities ranged from 0.41 to 0.46, implying acceptable validity and reproducibility. This measurement of LTPA has been used to examine the relationship between LTPA and self-rated health in Taiwanese adults with diabetes [24]. Physical activity measures using a similar format have been shown to be predictive of mortality in the general population and adults with diabetes [17,25]. In this study, the number of metabolic equivalents (METs) for each activity was calculated based on the report by Ainsworth et al. [26]. The participants were divided into 3 groups based on their LTPAs: (1) a regularly active group who reported ≧150 min/week of moderate-intensity activity (METs values between 3 and 6); (2) an intermediately active group who reported engaging in LTPA but did not meet the criterion for the “regular” category; and (3) an inactive group who reported no LTPA.

Basic demographic information (such as age, sex, education, body weight and height) was obtained from the questionnaires. Body mass index (BMI) was calculated as weight (kilograms) divided by height squared (meters squared). Other factors associated with mortality among people with diabetes, including the duration of diabetes, the use of insulin, health behaviours (including smoking), and the presence of comorbidities, were also considered. The list of comorbidities included a history of heart disease, hypertension, dyslipidaemia, or stroke. For each disease (including diabetes), participants were asked whether the diagnosis had been confirmed by a medical professional. A small number of respondents did not answer all of the questions. For example, two participants had missing data for education. This information is available in the form of a footnote below Table 1.

**Table 1:** Baseline characteristics of participants

	**Total**	**Deceased**	**Survived**	**P-value**^**b**^
N	701	121	580	
Age (years)	60.6 ± 0.44	66.2 ± 1.02	59.4 ± 0.47	<0.001
Sex (% female)	46.9	40.5	48.3	0.119
Education^a^ (%)				0.001
≥ 0 and < 7 years	59.7	73.6	56.7	
≥ 7 years	40.3	26.4	43.3	
Smoking (% current)	23.1	28.1	22.1	0.152
Body mass index^a^ (kg/m^2^)	25.1 ± 0.16	24.2 ± 0.45	25.2 ± 0.17	0.022
Duration of diabetes^a^ (years)	7.4 ± 0.29	11.6 ± 0.85	6.5 ± 0.29	<0.001
Using insulin^a^ (% yes)	14.2	21.5	12.7	0.011
Heart disease^a^ (% yes)	24.9	35.8	22.5	0.002
Hypertension^a^ (% yes)	44.3	57.5	41.6	0.001
Dyslipidaemia^a^ (% yes)	37.9	32.4	39.0	0.204
Stroke^a^ (% yes)	6.9	18.5	4.5	<0.001
Leisure time physical activity (%)				0.004
Inactive	43.5	56.2	40.9	
Intermediate	35.7	31.4	36.6	
Regular	20.8	12.4	22.6	
SF-36				
Physical function	75.1 ± 1.01	58.3 ± 2.76	78.6 ± 1.01	<0.001
Role: physical	59.7 ± 1.72	36.6 ± 4.19	64.5 ± 1.83	<0.001
Bodily pain index	69.3 ± 0.97	59.5 ± 2.47	71.3 ± 1.03	<0.001
General health perceptions	48.4 ± 0.78	41.0 ± 1.93	49.9 ± 0.84	<0.001
Vitality	57.6 ± 0.83	50.1 ± 1.96	59.2 ± 0.91	<0.001
Social functioning	77.9 ± 0.91	67.7 ± 2.57	80.1 ± 0.93	<0.001
Role: emotional	65.5 ± 1.66	54.5 ± 4.30	67.8 ± 1.78	0.005
Mental health index	68.4 ± 0.76	65.2 ± 1.89	69.0 ± 0.83	0.060
Physical component summary	40.3 ± 0.54	31.2 ± 1.45	42.2 ± 0.55	<0.001
Mental component summary	47.2 ± 0.43	46.5 ± 1.11	47.4 ± 0.47	0.450

^a^There were missing data.

^b^Continuous variables were compared using the Student’s *t*-test and shown as means ± standard errors. Categorical variables were compared using Pearson’s chi-square test and shown as a percentage of the total.

### Statistical analysis

We used the Student’s *t*-test or Pearson’s chi-square test to compare baseline characteristics between surviving and non-surviving participants. Cox proportional-hazards models were used to investigate the associations between baseline characteristics and mortality. The proportional hazards assumption (tested using Schoenfeld residuals) was not violated. Hazard ratios (HRs) and 95% confidence intervals (95% CIs) for mortality were estimated. In order to examine whether adults with diabetes with inferior HRQOL, combined with higher levels of LTPA had a reduced risk of mortality, joint HRQOL and LTPA variables were created. PCS and MCS scores were dichotomised (high vs. low) at the median (PCS = 45.11; MCS = 47.91). To study survival, Kaplan-Meier curves were plotted. We evaluated the discriminatory ability of PCS or LTPA or both to predict death by using the area under the receiver operating characteristic curve (C-statistics). We estimated C-statistics with a macro using R language which was developed based on Harrell’s C-statistics, and used these to evaluate the Cox regression models [27]. Apart from estimation of C-statistics, all analyses were conducted using SAS statistical software, version 9.1 (SAS Institute, Cary, NC), and SPSS statistical software, version 14.0 (SPSS, Chicago, IL., USA).

## Results

Of 701 participants aged 18 or older with diabetes, 121 participants died between baseline and 7-year follow-up, giving a crude mortality rate of 17.2% (121/701). Table 1 compares baseline characteristics between survivors and non-survivors. Surviving participants were significantly younger, had higher education levels, had a shorter duration of diabetes, were less likely to use insulin, were less likely to have a history of heart disease, hypertension or stroke, were more likely to engage in LTPA, and had a higher mean BMI, PCS, and scores for each subscale of the SF-36 (except for the mental health index and MCS).

In Table 2 and Table 3, we present three models that explore the relationships among PCS, MCS, LTPA and mortality. Model 1 was adjusted for age, sex, education, smoking, BMI, the duration of diabetes, insulin use, and any history of heart disease, hypertension, dyslipidaemia or stroke. Model 2 was additionally adjusted for either PCS or LTPA. The adjusted HR for mortality for a low vs. high PCS score was 2.15 (95% CI = 1.22-3.81) (Table 2, Model 1). This association remained statistically significant after additionally adjusting for LTPA (Table 2, Model 2). We then further categorized PCS and MCS scores by quartiles. After adjusting for other variables and LTPA, the adjusted HR for mortality for the lowest quartile group vs. the highest quartile group was 3.67 (95% CI = 1.61-8.37). A low score in the MCS was not significantly associated with increased mortality after adjusting for the other variables and additionally adjusting for LTPA. The adjusted HRs for mortality were 0.40 (95% CI = 0.22-0.71) in the intermediately active participants and 0.38 (95% CI = 0.19-0.76) in the regularly active participants compared with inactive participants (Table 3, Model 1). These associations remained identical after additionally adjusting for PCS scores (Table 3, Model 2). The C-statistics for the models ranged from 0.675 to 0.793, indicating good model prediction of mortality.

**Table 2:** Adjusted hazard ratios (HRs) and 95% confidence intervals (95% CIs) for mortality according to SF-36 physical component summary (PCS) and mental component summary (MCS) scores in Taiwanese adults with diabetes

	**N**	**Number of deaths**	**Crude death rate (%)**	**Age-adjusted**			**Model 1**			**Model 2**		
				**HR (95% CI)**	**P-value**	**C-statistic**	**HR (95% CI)**	**P-value**	**C-statistic**	**HR (95% CI)**	**P-value**	**C-statistic**
PCS						0.706			0.779			0.793
High (≥ 45.11)	351	33	9.4	Reference			Reference			Reference		
Low (< 45.11)	350	88	25.1	2.19 (1.44-3.34)	<0.001		2.15 (1.22-3.81)	0.008		2.17 (1.22-3.85)	0.008	
PCS quartile groups												
≥ 51.92	175	17	9.7	Reference			Reference			Reference		
≥ 45.11, <51.92	176	16	9.0	0.81 (0.41-1.61)	0.545		0.96 (0.38-2.41)	0.935		1.02 (0.41-2.56)	0.961	
≥ 30.06, <45.11	175	24	13.7	1.16 (0.61-2.18)	0.653		1.15 (0.48-2.76)	0.748		1.30 (0.54-3.10)	0.559	
<30.06	175	64	36.5	3.03 (1.69-5.41)	<0.001		3.82 (1.67-8.75)	0.001		3.67 (1.61-8.37)	0.002	
MCS						0.675			0.764			0.785
High (≥ 47.91)	351	57	16.2	Reference			Reference			Reference		
Low (< 47.91)	350	64	18.2	1.15 (0.80-1.64)	0.457		1.34 (0.83-2.18)	0.236		1.23 (0.75-2.02)	0.406	

PCS and MCS were in separate models in all of the analyses. Model 1 was adjusted for age, sex, education, smoking, BMI, the duration of diabetes, insulin use, and any history of heart disease, hypertension, dyslipidaemia, or stroke. Model 2 was also adjusted for LTPA.

**Table 3:** Adjusted hazard ratios (HRs) and 95% confidence intervals (95% CIs) for mortality according to levels of leisure time physical activity in Taiwanese adults with diabetes

	**N**	**Number of deaths**	**Crude death rate (%)**	**Age-adjusted**			**Model 1**			**Model 2**		
				**HR (95% CI)**	**P-value**	**C-statistic**	**HR (95% CI)**	**P-value**	**C-statistic**	**HR (95% CI)**	**P-value**	**C-statistic**
LTPA						0.697			0.784			0.793
Inactive	305	68	22.2	Reference			Reference			Reference		
Intermediate	250	38	15.2	0.60	0.011		0.40	0.002		0.40	0.002	
				(0.40-0.89)			(0.22-0.71)			(0.23-0.72)		
Regular	146	15	10.2	0.41	0.002		0.38	0.006		0.38	0.006	
				(0.23-0.71)			(0.19-0.76)			(0.19-0.76)		

Model 1 was adjusted for age, sex, education, smoking, BMI, the duration of diabetes, insulin use, and any history of heart disease, hypertension, dyslipidaemia, or stroke. Model 2 was also adjusted for PCS.

Table 4 compares baseline characteristics between those with high and low PCS scores after stratifying by level of LTPA. The results show that regardless of LTPA level, participants with low PCS scores were significantly older, had lower educational levels, had a longer duration of diabetes, and had a greater prevalence of hypertension than participants with high PCS scores.

**Table 4:** Characteristics of participants according to SF-36 physical component summary scores (PCS) and stratified by level of leisure time physical activity

	**Leisure time physical activity**								
	**Inactive**			**Intermediate**			**Regular**		
	**PCS**	**PCS**		**PCS**	**PCS**		**PCS**	**PCS**	
	**(High)**	**(Low)**		**(High)**	**(Low)**		**(High)**	**(Low)**	
			**P-value**^**a**^			**P-value**^**a**^			**P-value**^**a**^
N	148	157		124	126		79	67	
PCS									
First quartile (Q1)	49.1	17.3		49.0	21.9		50.0	23.7	
Second quartile (Q2)	52.0	28.0		51.6	31.2		52.5	32.9	
Third quartile (Q3)	54.3	36.2		54.5	38.8		54.4	40.2	
Age (years)	53.8 ± 0.83	65.2 ± 0.89	<0.001	59.3 ± 1.03	64.1 ± 0.96	0.001	57.7 ± 1.25	63.6 ± 1.30	0.001
Sex (% female)	39.9	54.8	0.009	50.8	54.0	0.617	30.4	43.3	0.106
Education			<0.0001			0.042			<0.001
≥ 0 and <7 years	57.5	80.3		51.6	64.3		27.8	59.7	
≥ 7 years	42.5	19.7		48.4	35.7		72.2	40.3	
Smoking (% current)	37.2	22.3	0.004	19.4	14.3	0.284	22.8	17.9	0.468
Body mass index (kg/m^2^)	24.9 ± 0.30	25.3 ± 0.42	0.491	25.2 ± 0.37	25.1 ± 0.37	0.768	25.1 ± 0.39	25.0 ± 0.54	0.899
Duration of diabetes (years)	5.1 ± 0.45	9.0 ± 0.71	<0.001	6.5 ± 0.65	9.1 ± 0.74	0.008	5.6 ± 0.58	9.1 ± 1.11	0.006
Using insulin (% yes)	12.2	22.6	0.017	8.9	14.3	0.181	6.4	17.9	0.032
Heart disease (% yes)	14.6	34.7	<0.001	11.8	37.6	<0.001	19.0	31.3	0.084
Hypertension (% yes)	34.0	51.0	0.003	33.9	57.6	<0.001	36.7	55.2	0.025
Dyslipidaemia (% yes)	32.4	37.9	0.338	40.0	39.5	0.935	28.9	54.0	0.003
Stroke (% yes)	2.0	9.7	0.005	1.6	16.8	<0.001	2.5	7.5	0.165

^a^Continuous variables were compared using the Student’s *t*-test and shown as means ± standard errors. Categorical variables were compared using Pearson’s chi-square test and shown as a percentage of the total.

**Figure 1:**
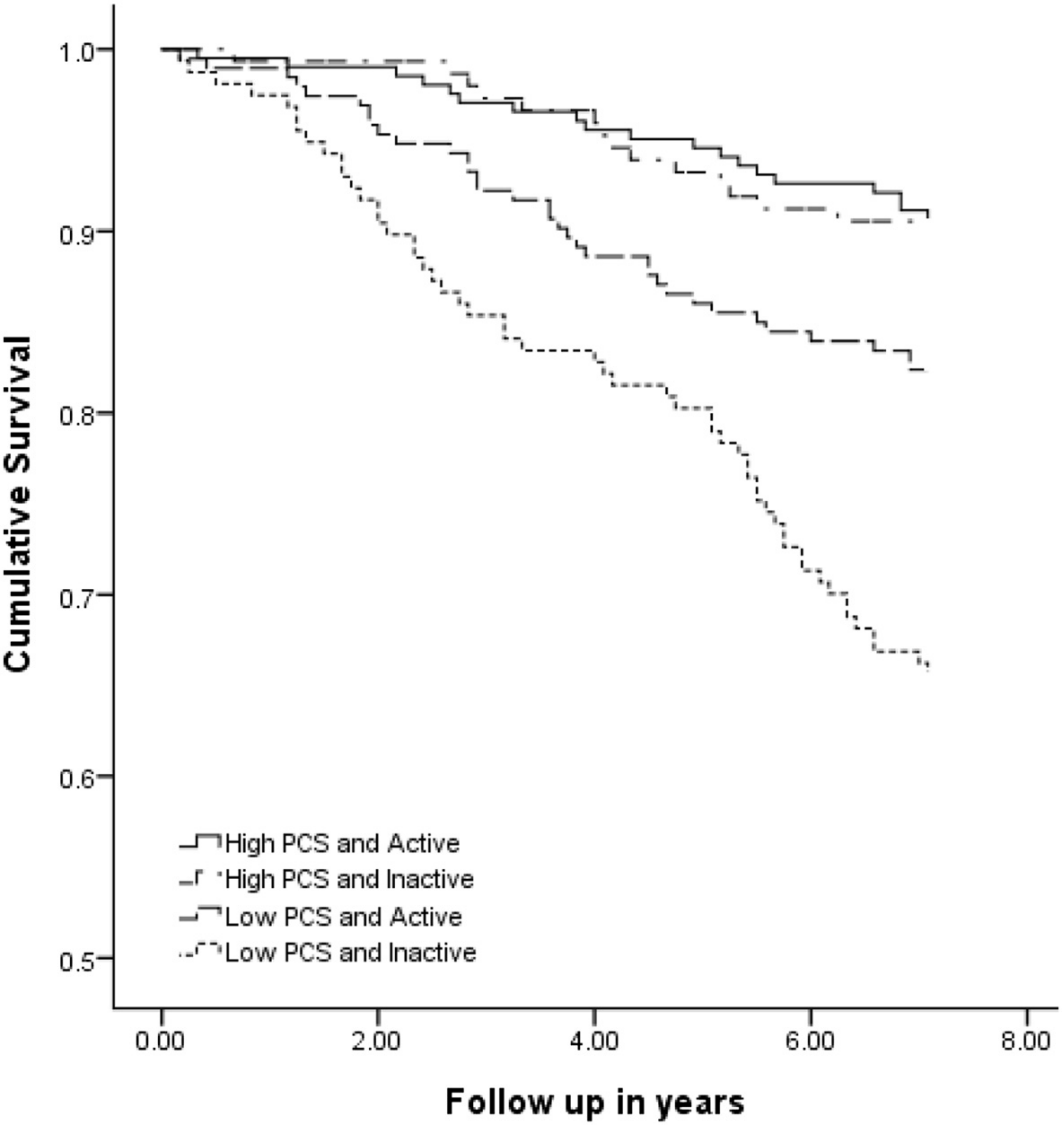
Survival curves according to categories of levels of LTPA and scores for the PCS of the SF-36.

Figure 1 shows the survival curves for participants according to categories of levels of LTPA and scores for the PCS of the SF-36. We have further classified LTPA into active (including intermediate and regularly active) versus inactive groups. Table 5 shows the combined associations of LTPA and PCS with mortality. After adjusting for other variables, those who were inactive and had a low PCS score had significantly higher mortality (HR = 4.49; 95% CI = [2.15-9.36]; P-value < 0.001) than those who were active and had a high PCS score.

**Table 5:** Adjusted Hazard Ratios (HR) and 95% Confidence Intervals (95% CI) for mortality by levels of leisure time physical activity and scores for the physical component summary (PCS) of the SF-36 among people with diabetes in Taiwan

**Variable**	**N**	**Number of deaths**	**Crude death rate (%)**	**Age-adjusted**^**a**^		**Multivariate adjusted**^**b**^	
				**HR (95% CI)**	**P-value**	**HR (95% CI)**	**P-value**
PCS and LTPA							
High PCS and Active^c^	203	19	9.3	Reference		Reference	
High PCS and Inactive	148	14	9.4	1.25 (0.62-2.50)	0.537	1.31 (0.51-3.35)	0.578
Low PCS and Active^c^	193	34	17.6	1.65 (0.94-2.91)	0.083	1.36 (0.64-2.92)	0.426
Low PCS and Inactive	157	54	34.3	3.38 (1.98-5.75)	<0.001	4.49 (2.15-9.36)	<0.001
C-Statistic				0.722		0.792	

^a^Cox proportional hazard models were used to estimate the HR adjusted for age.

^b^Cox proportional-hazards models were used to estimate the HR adjusted for age, sex, education, smoking, BMI, the duration of diabetes, insulin use, and any history of heart disease, hypertension, dyslipidaemia, or stroke.

^c^Active group includes participants who were intermediately or regularly active.

## Discussion

We demonstrated that both PCS scores and LTPA predict mortality in individuals with diabetes over 7 years of follow up. Furthermore, our study confirms that low PCS scores are associated with increased mortality in adults with diabetes, even after controlling for LTPA and other potential confounders. Our study further revealed that only participants who reported no LTPA with low PCS scores had significantly increased mortality compared to active participants with high PCS scores. Most importantly, there was no excess mortality risk associated with low PCS scores in participants who engaged in an intermediate or regular amount of leisure activity. Our joint analyses broaden our understanding of the benefits of engaging in LTPA, which may be associated with improved survival in participants with poor self-rated physical health status.

In this study, participants with low PCS scores were significantly older, had lower educational levels, had a longer duration of diabetes, and had a higher prevalence of hypertension than participants with high PCS scores, independent of LTPA level (Table 4). Previous studies have consistently shown that older people with diabetes have considerable physical impairment [28,29]. Our recent study has further shown that there is a gradient effect of functional decline on mortality in older adults with diabetes [30]. Glasgow et al. found that individuals diagnosed with diabetes for a greater number of years reported lower physical functioning (as assessed by the SF-20) than those with fewer years since diagnosis [31]. Longer diabetes duration is associated with an increased risk of exposure to chronic hyperglycaemia and an increased prevalence of cardiovascular disease such as hypertension, dyslipidaemia, and heart disease which may be associated with elevated mortality risk [14]. In a prospective cohort study, de Visser et al. found that a decreased score on the dimension of physical functioning (as assessed by the RADN-36) in individuals with type 2 diabetes is associated with short-term onset of cardiovascular disease [32]. Prior research has also found that patients with diabetes who report more bodily pain and poor physical functioning (as assessed by the SF-36) and poorer self-reported overall health are more likely to have an elevated glycosylated haemoglobin [33]. Most past research agrees that a higher glycosylated haemoglobin level, a measure of disease control in diabetes, is an important risk factor for mortality in people with diabetes. Diabetes complications such as coronary artery disease have a significant negative impact on the physical domains of HRQOL (as assessed by the SF-36) in individuals with diabetes [10]. These abovementioned research findings could explain the possible mechanisms behind our observed association between a reduced score on the physical aspect of HRQOL and increased mortality in individuals with diabetes.

Prior studies agree that increased LTPA is associated with reduced mortality in individuals with diabetes via several mechanisms. It has been suggested that engagement in regular physical activity may improve insulin sensitivity, glycemic control, cardiorespiratory fitness and physical function, as well as having favourable effects on hypertension and serum lipid profile [15,17,34-36]. Using a randomized controlled trial, Balducci et al. demonstrated that even mild aerobic exercise training, such as brisk walking, can modify the natural history of peripheral neuropathy in both type 1 and type 2 diabetes patients [37]. Thus, although factors such as an older age, long duration of diabetes, hyperglycaemia, cardiovascular disease, and diabetes complications may contribute to reduced scores on the physical health domains of HRQOL, the benefits of becoming physically active may translate into protective effects against their impacts on mortality. This is supported by our finding that in participants who engaged in intermediate or regular leisure time activity, no significant association was observed between low PCS scores and mortality (Table 5). Further investigation is needed to explore the underlying obstacles to engaging in LTPA and the underlying causes of increased mortality in adults with diabetes with inferior physical health domains of HRQOL. Moreover, intervention studies are needed to assess whether such individuals would benefit from interventions aimed at increasing their engagement in LTPA and improving survival.

Our finding that MCS scores did not predict increased mortality in individuals with diabetes is consistent with two previous studies [1,2]. In the first study carried out in the Zwolle region of the Netherlands, no association was observed between MCS scores and mortality in individuals with type 2 diabetes over a median follow-up period of 5.8 years [1]. However, the same investigators revisited the association between HRQOL and mortality after a longer follow-up period (almost 10 years), and found that low MCS scores were associated with higher mortality in patients with type 2 diabetes, suggesting that lower MCS scores only predict mortality after a longer follow-up period [38]. These findings may help explain why our results revealed no association between MCS scores and mortality in individuals with diabetes over only a 7-year follow-up period.

Our study has several limitations. Although the initial sample of 797 individuals with self-reported diagnosed diabetes is a nationally representative sample of adults aged 18 years or older with diagnosed diabetes, our analytic sample could be biased due to the included participants being limited to those who had complete data for HRQOL and LTPA. The comparison of characteristics between respondents who were included (N = 701) and excluded due to incomplete data (N = 93) from this study suggests that our sample could be biased towards individuals of younger age, with a shorter duration of diabetes, and who are less likely to be female, not smoking currently, and have a history of hypertension and stroke. Thus, some care should be taken when generalizing our results to the whole population. In addition, individuals with incomplete data for HRQOL or LTPA who were excluded from this study had a significantly higher mortality than individuals included in the study. Therefore, the observed crude mortality rate of 26.5 per 1,000 person-years may be an underestimate and the association of HRQOL and LTPA with mortality may also be underestimated. As we were still able to demonstrate a statistically significant association between HRQOL and LTPA and mortality despite this potential underestimation, it is likely that the association between HRQOL and LTPA and risk of mortality among adults with diabetes is substantial.

Our analyses focused on LTPA because it was the only type of physical activity measure included in the 2001 NHIS and as a result, we may have underestimated the total physical activity levels of our participants. It is possible that some participants classified into the inactive group because they reported no LTPA, might be performing other types of physical activity such as housework leading to misclassification. The relationships between HRQOL and LTPA and mortality could differ by duration of diabetes and age. However, we were unable to investigate the impact of these factors on the association between HRQOL and LTPA and mortality as the small size of our sample did not enable us to stratify our participants by these characteristics when investigating the associations between HRQOL and LTPA and mortality. We did not control for clinically relevant variables such as glycated haemoglobin levels, urinary albumin-to-creatinine ratio, systolic blood pressure, serum creatinine, diabetes complications, and antidepressants. Therefore, residual confounding by these factors may have affected our results. As this study was an observational study, we cannot confirm the causal relationship between LTPA and HRQOL and mortality. It is very likely that the presence of severe health issues which precluded LTPA are also leading to reduced HRQOL and increased mortality. A sensitivity analysis excluding deaths that occurred during the first 2 years of follow-up would help correct for the impact of severe health issues at the start of the study, which could have an influence on both activity levels and HRQOL, in addition to an influence on mortality. After excluding deaths that occurred during the first 2 years of follow-up from the analysis, our finding of an association between increased LTPA and reduced mortality risk in adults with diabetes and inferior HRQOL remained unchanged. No data were available regarding the type of diabetes, and it is possible that HRQOL could vary based on diabetes type. However, only a small proportion (<3%) of Taiwanese patients with diabetes have type 1 diabetes, and, therefore, it is likely that our results largely reflect the interrelationship between HRQOL, LTPA and the risk of mortality in patients with type 2 diabetes.

The major strength of our study is that we used a national sample of the Taiwanese population via the NHIS data and the National Register of Deaths. As this study was an observational study, we cannot rule out the possibility that the presence of poor self-rated HRQOL leads to lesser or no LTPA. However, we believe that this is unlikely to be the case in our study, because among participants with low PCS the percentage of those who reported regular leisure activities (19.1%; 67/350) was not significantly different from that in participants with high PCS (22.5%; 79/351). It is worthwhile noting that a great proportion of participants with diabetes reported no LTPA, regardless of either good or poor self-rated physical health (42.2% vs. 44.9%). This result may reflect a general undervaluing of the merits of LTPA by individuals with diabetes. Our data further suggest that health care professionals should particularly target interventions aimed at improving survival towards adults with diabetes who are more likely to have reduced physical HRQOL, such as those who are older, have lower educational levels, have a longer duration of diabetes, and have a history of hypertension (Table 4).

## Conclusions

To our knowledge, this is the first study to assess the interrelationship between physical health domains of HRQOL, LTPA and the risk of mortality in individuals with diabetes. Our results have important practical implications. Our finding of a C-statistic for the model based on joint PCS scores and LTPA variables and age of 0.722 suggests that the combined use of PCS scores and LTPA may be useful in identifying individuals with diabetes who are at higher risk of mortality in the absence of laboratory and clinical indicators. Our data suggest that health care professionals should pay careful attention to patient-reported HRQOL and levels of LTPA. Our results also suggest that health care professionals should be aware of the possible need for more care in individuals who report no LTPA to reduce the mortality risk of diabetes associated with reduced HRQOL in these persons. There is increasing recognition of the importance of patient-reported HRQOL as a part of individualized care in adults with diabetes. More research is needed to evaluate how HRQOL information can be used for health care planning and to inform resource allocation decisions. Our findings add to our understanding of the potential benefit of LTPA, even at levels not meeting diabetes-specific guidelines (i.e., 150 minutes of moderately intense activity per week), in terms of reducing the excess mortality associated with poor physical health. We hope our findings will stimulate further efforts to design and implement programs aimed at increasing LTPA, especially in individuals with diabetes who have poor self-rated physical health.

## Abbreviations

HRQOL: Health Related Quality Of Life; LTPA: Leisure-Time Physical Activity; NHIS: National Health Interview Survey; SF36: Short Form-36; PCS: Physical Component Summary; MCS: Mental Component Summary; HR: Hazard Ratios; CI: Confidence Interval; METs: Metabolic Equivalents; BMI: Body Mass Index.

## Competing interests

None of the authors report any conflicts of interests.

## Authors’ contributions

CLL initiated the study, reviewed the data, and drafted and revised the manuscript. HYC conducted the NHIS survey, carried out the data analysis, reviewed the data, discussed the study, and revised the manuscript. CCH and JFRL reviewed the data, discussed the study and provided valuable comments on the manuscript. HLF carried out the data analysis, reviewed the data, and discussed the study. All authors reviewed the manuscript and approved the final version.

## Pre-publication history

The pre-publication history for this paper can be accessed here:

http://www.biomedcentral.com/1471-2458/13/67/prepub
